# Modeling Adaptation Strategies against Climate Change Impacts in Integrated Rice-Wheat Agricultural Production System of Pakistan

**DOI:** 10.3390/ijerph17072522

**Published:** 2020-04-07

**Authors:** Muhammad Khalid Anser, Tayyaba Hina, Shahzad Hameed, Muhammad Hamid Nasir, Ishfaq Ahmad, Muhammad Asad ur Rehman Naseer

**Affiliations:** 1 School of Public Administration, Xi’an University of Architecture and Technology, Xi’an 710055, China; mkhalidrao@xauat.edu.cn; 2 Institute of Agricultural and Resource Economics, University of Agriculture Faisalabad, Punjab 38040, Pakistan; 3 The School of Economics and Finance, Xi’an Jiaotong University, Xi’an 710049, China; shahzadimit@gmail.com (S.H.); cuteecono95@stu.xjtu.edu.cn (M.H.N.); 4 Centre for Climate Research and Development, COMSATS University, Islamabad 45550, Pakistan; ishfaqahmad@comsats.edu.pk

**Keywords:** rice-wheat agricultural system, climate change, impact assessment, adaptation packages, Pakistan

## Abstract

There are numerous anticipated effects of climate change (CC) on agriculture in the developing and the developed world. Pakistan is among the top ten most prone nations to CC in the world. The objective of this analysis was to quantify the economic impacts of CC on the agricultural production system and to quantify the impacts of suggested adaptation strategies at the farm level. The study was conducted in the Punjab province’s rice-wheat cropping system. For this purpose, climate modeling was carried out by using two representative concentration pathways (RCPs), i.e., RCPs 4.5 and 8.5, and five global circulation models (GCMs). The crop modeling was carried out by using the Agricultural Production Systems Simulator (APSIM) and the Decision Support System for Agrotechnology Transfer (DSSAT) crop simulation models (CSMs), which were tested on the cross-sectional data of 217 farm households collected from the seven strata in the study area. The socio-economic impacts were calculated using the Multidimensional Impact Assessment Tradeoff Analysis Model (TOA-MD). The results revealed that CC’s net economic impact using both RCPs and CSMs was negative. In both CSMs, the poverty status was higher in RCP 8.5 than in RCP 4.5. The adaptation package showed positive results in poverty reduction and improvement in the livelihood conditions of the agricultural households. The adoption rate for DSSAT was about 78%, and for APSIM, it was about 68%. The adaptation benefits observed in DSSAT were higher than in APSIM. The results showed that the suggested adaptations could have a significant impact on the resilience of the atmospheric changes. Therefore, without these adaptation measures, i.e., increase in sowing density, improved cultivars, increase in nitrogen use, and fertigation, there would be negative impacts of CC that would capitalize on livelihood and food security in the study area.

## 1. Introduction

Climate change is real, and its observed effects on physical and biological systems have been negative over time. Previous literature also demonstrated the disastrous impacts of climate change on ecological and social systems [1,2,3,4,5,6,7,8]. Overtime and mean changes in climate factors such as temperature, precipitation, and sea level are observed across the globe. The impact of climate change on the intensity and the frequency of disasters such as hurricanes, droughts, storms and cyclones, fires, heatwaves, and epidemics vary around the world, depending on the current geographic climate conditions [9,10,11,12,13]

Agricultural activities and security in production depend mainly on environmental factors, e.g., temperature, precipitation, and wind [14,15]. In developing countries, aggravated climate change has major impacts on the livelihood and the food security of poor households living in rural areas who are largely dependent on the agricultural production system [16,17,18]. This threatens the status of food security and poverty around the globe, because farming is important to ensure these two most important issues. The countries in South Asia are the most affected by climate change, and agriculture is the largest part of the gross domestic product (GDP) in these economies, posing serious challenges to social and economic conditions of the region [19,20,21]. 

In vulnerable regions of the world, climate-related factors are strong predictors of crop and animal production, income, disease, and undernutrition [22,23]. Approximately 65% of Pakistan’s population is living in rural areas, and their livelihoods are directly or indirectly dependent on the agricultural sector. Agriculture contributes 18.6% to the national GDP, and it employs about 42% of the country’s workforce [24]. Even with the high share of agriculture in the economy of Pakistan, no efforts have been made to address the alarming situation of climate-induced problems and extreme weather events in this sector [25,26]. Pakistan is one of the most affected countries due to climate change in South Asia and around the world. In the period 1998–2012, the Global Climate Risk Index (GCRI) and the World Bank report put Pakistan in seventh place in the index of countries facing climate extremes [27]. 

Uncertainty in climate impacts assessment includes the complexity of atmospheric models, downscaling methods, greenhouse gas emission scenarios, and uncertainty in crop models [28,29,30]. Most of the studies used single emission scenarios, climate models that cannot characterize the climate risk [31,32,33,34]. More robust climate change impact assessment is based on multi-climate model global circulation models (GCMs) projections as well as multiple scenarios. The process-based crop models are used for climate impact assessment and to reduce the uncertainty associated with climate scenarios [35].

Adaptation can manage the negative climate change impacts, but it cannot solve the climate change problem on its own. Adaptation is referred to as changes in the human–environment mechanism concerning current and predicted changing climatic conditions to minimize or neutralize the associated risks that generate potential opportunities against climate change [36]. Adapting the agricultural production system to climate change is critical in developing countries to ensure the livelihoods of poor communities [3]. Adaptation strategies are effective to help the residents to cope with severe weather and climate change events [7,37,38,39,40]. 

The strategies for adapting climate are context-specific and site-specific and evolve in dependent communities [40,41]. The factors responsible for adaptive response variability across regions include all the agro-ecological systems, socioeconomics, environmental effects, and existing infrastructure and capability [14,42,43,44]. Multiple stakeholders should be involved in building adaptation strategies, including policymakers, researchers, non-profit organizations (NGOs), communities, extension agents, and farmers. These adaptations are predominantly location-specific and depend on local institutions and socioeconomic conditions of the dependents [45]. Adaptation research needs to be solution-oriented due to the impending existence of environmental problems. Adaptations help to address climate change, but there is considerable uncertainty about the impacts and the adaptation effectiveness [38,46].

Generally, small and poor farmers across the globe and specifically in the South Asian region have limited capabilities to invest in management practices and technology adoption [47]. The climate instability coupled with other critical socio-economic pressures, e.g., being unable to buy and apply inputs at required prices or within a reasonable timeframe, poor management of crops due to shortages of labor, or competing for off-farm opportunities of livelihoods, farmers can be trapped in a cycle of low adaptability and, therefore, vulnerability to climate change [48,49]. In contrast, adaptations are observed by farmers in South Asia who experience and strive to tackle climate variations such as different crop species selection, management practices, sowing dates, and use of irrigation to overcome drought and heat stress [50,51]. Not all farmers, however, are equally capable of responding to climate fluctuations, particularly if the temperature or the precipitation change significantly. This highlights the complexity and the risks associated with the cultivation of smallholders’ crops in tropical and subtropical climates, a condition that needs mitigation to achieve sustainable growth [52,53]. 

The subsistence of resource-poor rural households is largely dependent on agriculture in developing countries such as Pakistan, but this sector of the economy is the most vulnerable to climate change and volatility. The households’ capacity to adapt to climate change effects, which threaten households’ resources and sustainability, is uncertain due to poor socioeconomic conditions of the agricultural community [2,8,54,55,56]. Rice and wheat are the two main staple cereal crops of Pakistan and are grown under various climatic and hydrological conditions throughout all agroecological zones of the country [8]. The concerns about the productivity of rice and wheat crops are very important, because these two crops contribute approximately 20% and 75% of Pakistanis’ average daily calorie intake [57].

Pakistani rice and wheat yield varied in the first decade of the millennium by up to 1.31 and 0.57 tons per hectare at the national level [58,59]. The socio-economic conditions of smallholder farmers are prone to environmental and some other non-climatic threats to agriculture. Several studies were carried out to measure the climate impact threats on the integrated rice-wheat cropping zone in Pakistan, which showed that climate change has adverse effects on both crops’ productivity [8,47,60]. Climate change impact assessment needs to be investigated in all agro-ecological areas, and there is a need for future adaptations to national climatic conditions. It also needs to be redefined to minimize the dangerous impacts of climate change.

There is a need for different climate adaptation strategies in collaboration with other science disciplines, extension and support services, and stakeholder’s engagement for rice and wheat crops. Therefore, this study focused on assessing climate change sensitivity to Pakistan’s rice-wheat agricultural production system and also incorporated the impact assessment of the planned climate change adaptation package at the farm level. The adaptation package was drawn up following the needs of both crops. The main features of these strategies were to change the sowing date patterns, modification of the plant population, adoption of new sowing and harvesting technologies, and modernized irrigation and fertilizer application methods.

## 2. Materials and Methods

### 2.1. Description of the Study Area

**Figure 1:**
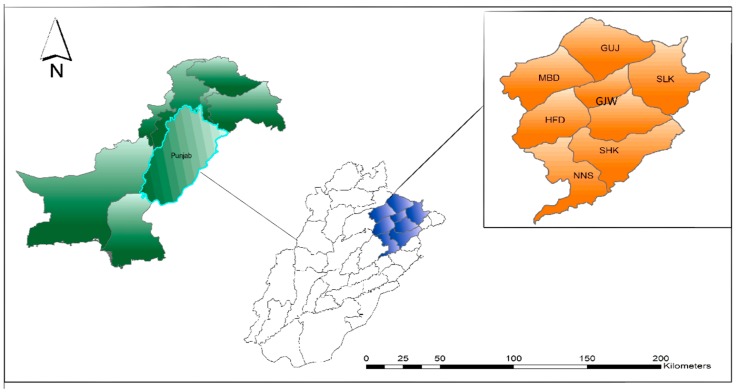
Study area map.

In Pakistan, Punjab is the most populated province and contributes a major share in the national agriculture production. There are five major agro-ecological zones in the province of Punjab-Pakistan, i.e., the rice-wheat zone, the cotton-wheat zone, the mixed-cropping zone, the low-intensity zone, and the rain-fed zone. The rice-wheat zone was purposely chosen for the study because of its importance in ensuring the food security of the country and the importance of export revenue earned through both crops. The rice-wheat cropping system is the major one that accounts for a total of 2.2 million ha of area, supporting the livelihood of 1.1 million farm families [61]. In Pakistan, the rice-wheat cropping areas are mainly located in central Punjab. Main districts include Sheikhupura (SHK), Nankana Sahib (NNS), Hafizabad (HFD), Gujranwala (GJW), Sialkot (SLK), Gujrat (GUJ), and Mandi Bahauddin (MBD). The study covers the Rice Wheat Cropping System (RWCS) of Punjab province comprising the seven famous strata mentioned earlier and forming a heterogeneous sample size, as shown in Figure 1.

### 2.2. Collection of Farm Surveyed Data

Both primary and secondary data were collected and used in this study. Primary data were collected from farmers after taking their consent to provide information using a well-structured questionnaire. For secondary data, different government sources and surveys, i.e., Soil Surveys, Economic Surveys of Pakistan, the Pakistan Meteorological Department, and the Pakistan Bureau of Statistics were used.

The agricultural population is heterogeneous; therefore, the methodology of multi-stage stratified random sampling was used to collect the primary data following Naseer et al. [62]. In the first stage, the RWCS was chosen purposely for this study due to its importance of both major crops, rice and wheat, used as a staple food. In the second stage, all the seven rice-producing districts were chosen from the RWCS, i.e., Sheikhupura, Nankana Sahib, Hafizabad, Gujranwala, Sialkot, Gujrat, and Mandi-Bahauddin, which form the seven strata of the study. In the third stage, three villages from each stratum were chosen randomly. In the last stage, ten respondents from each village were chosen randomly, which made the total sample size of 210 respondents. All ethical considerations and anonymity of the respondents were considered and assured to the respondents. Participation of the respondents in the sample size was on a volunteer basis. The study aims and objectives were explained to all respondents before data collection. Prior to data collection, training of the data collection team was carried out. Data were collected by the students of Ph.D. and master’s degrees at the University of Agriculture Faisalabad, Pakistan.

### 2.3. Climate Change Projections

A baseline daily weather dataset (1980–2009) was produced by using a well-developed climatic methodology following the Coupled Model Intercomparison Project (CMIP5) [63]. Historical (baseline) weather information from observations of local stations is the basis for model inter-comparison and initial study of climate information and calibration of crop models. In the study area, a total of 4 weather stations (Nankana Sahib, Sheikhupura, Sialkot, and Mandi Bahauddin) were identified in four different strata, in which all stations had a 30 year measured daily weather data. However, the 30 year daily weather data from the AgMIP Hybrid Baseline Climate Datasets (AgMERRA) for the remainder in three strata (Hafizabad, Gujranwala, and Gujrat) were created following Ruane et al. [64]. NASA Modern Era-Retrospective Analysis for Research and Analysis (MERRA) was used to generate daily maximum and minimum temperature, solar radiation, and precipitation for sites where it was impossible to collect physical weather data [65].

In this analysis, forecasts were made in CMIP5 emission scenarios for the mid-century (2040–2069) duration under RCP 4.5 (mild climatic conditions) and RCP 8.5 (harsh climatic conditions). Using the delta method, future scenarios were developed. The rationale for selecting two RCPs, 4.5 and 8.5, was the correspondence to the greenhouse gas (GHG) emissions scenario. RCP 4.5 reflects low GHG emissions and controlled demand for energy as well as the existence of climate change initiatives, while RCP 8.5 has the highest GHG emissions owing to rising demand for energy and a lack of climate change policies [66,67]. Statistical downscaling and climate change scenarios were produced by Pakistan Metrological Department (PMD), a method described by Ruane et al. [68]. The carbon dioxide concentration of 499 ppm was used for RCP 4.5 and 571 ppm for RCP 8.5 [69].

Using GCMs representing physical processes in the atmosphere, the ocean, the cryosphere, the and land surface, future climate scenarios were developed. GCMs are currently the most advanced tools available to simulate the global climate system’s response to increased concentrations of greenhouse gases. For this study, the five best GCMs were used. These models were the same for both RCPs (4.5 and 8.5) and are namely, BCC-CSM (cool wet), CCSM4 (cool dry), BNU-ESM (middle), CMCC-CM (hot dry), and MIROC-ESM (hot wet). The selected GCMs were downscaled using a delta method described by Ruane, et al. [68]. 

### 2.4. Crop Modeling 

In this study, two famous crop simulation models (CSMs)—the Agricultural Production Systems Simulator (APSIM) [70] version 7.5 and the Decision Support System for Agrotechnology Transfer (DSSAT) [71,72] version 4.6—were used. The genetic coefficients of models were estimated for rice and wheat cultivars with two years of field experimental data. Models were calibrated with first-year field data and evaluated with the second year. The data on phenology (days to anthesis, maturity), growth (leaf area index and total dry matter accumulation), yield, and biomass were collected for model parametrization. Rice cultivars Super Basmati, Basmati 380, and Basmati-2000 were used in this analysis, and wheat cultivars Sahar-06, Lasani-08, and Faisalabad-08 were calibrated and evaluated. The calibration and the evaluation followed procedures published previously, such as Ahmad et al. [73].

After the model’s parametrization, the crop models were evaluated with farmer surveyed data. Surveyed information on farmers’ fields included the wheat and rice cultivars that were grown, initial conditions (previous crop sown, remaining crop, and root residue weight), and crop management (sowing time, tillage, fertilizer and irrigation amounts, harvesting date, etc.). This farm management and soil series data were used to create input files for DSSAT and APSIM using the QUADUI tool. The crop models were run with one-year climate data, and the observed and the simulated yields were compared.

The sensitivity of models was estimated using carbon, temperature, water, and nitrogen (CTWN) analysis. The detailed description is given by Ahmad et al. [74]. 

For climate impact assessment, current and future climate scenarios under RCP 4.5 and 8.5, farm surveyed, and soil series data of each stratum were used as input in DSSAT and APSIM models. The models were run with 30 years of current and future climate data. 

Results from the crop model simulation provided insight into the biophysical effect in terms of yield changes. Such findings were then incorporated into an approach to farming systems to evaluate the impacts of climate change and the proposed adaptation package on the livelihoods of smallholder farmers. The next section describes the use of an economic impact assessment model using crop simulation outputs along with data from household survey data (e.g., farm size, off-farm income) and other secondary information to quantify vulnerability and possible climate change gains and losses under current and future conditions.

### 2.5. Economic Modeling

Economic assessment of climatic change sensitivity was done with the Tradeoff Analysis Model for Multidimensional Impact Assessment (TOA-MD) version 6.1 in this study [75,76,77]. The economic analysis was done on a per farm basis. All farm-based activities—major crops (rice and wheat), minor crops (fodder), and livestock—were included for the true representation of the existing socioeconomic conditions of the farming community of the surveyed farms. The analysis was done for both CSMs (APSIM and DSSAT) and both RCPs (RCP 4.5 and RCP 8.5) for each GCM simulation separately. The TOA-MD used simulated crop yields, price, and production patterns from global economic models [76,77,78]. It also provided an assessment of the potential benefits of a future adaptation strategy.

This economic model measures the proportion of households susceptible to climate change-related losses, subsequent gains and losses, as well as poverty rates and shifts in per capita income due to climate change. In the case of planned adaptation packages or technology initiatives, it often estimates a potential adoption rate. The participation of stakeholders directed the definition and the development of adaptation strategies. In this study, we assumed that the consequences of climate change on non-modeled crops (sorghum and maize) and livestock were dependent on secondary information. Livestock is an important part of the system in this region.

To define the current production system, we used survey data and other secondary information, including all economic activities in the farm (e.g. crop, livestock, household). Subsequently, we used crop simulations to define changes in yields due to climate change or crop management to assess the potential impacts on farm livelihoods, which we discuss further below.

The model considers farmers as economically rational beings to make decisions on the predictable value, and that is why it uses binary codes [79]. The farmers may choose to stick to system 1, or they can choose the alternative system 2. Normally, system 1 is described as the current production system with base production technology and the current climate, whereas system 2 is described as the current production system with base technology and the changing climate. The productivity of the system depends largely on two factors, technology and climate. Farmers’ decisions of whether to operate in system 1 or system 2 depend upon the opportunity cost (gains/losses) from switching.

(1) $$\omega =v_{1}-v_{2}$$

Net returns from systems 1 and 2 are shown in Equation 1, v1 and v2, respectively. The cost of the opportunity fits the distribution of ω. Farmers adopt system 2 if < 0, i.e., system 2 net income is higher than system 1 net income. Alternatively, they must stay in system 1 (non-adopters) if > 0, i.e., system 2 net returns are lower than system 1 net returns.

The poverty line was set to USD 1.25/person/day (USD 1 = RS 103) (RS: Pakistani rupee, it can also be written as PKR) in the analysis according to international standards, which were to check the vulnerability level of households with respect to climatic changes [80]. For climate change impact assessment (CC-IA) analysis, all the prices of inputs/outputs were site-specific according to the production system(s), and net returns were evaluated accordingly.

The model parameters of TOA-MD for systems 1 and 2 were determined by every farmer in data for the current time period following Valdivia et al. [81].

(2) $$\mu_{j}\left(T_{HH},\gamma_{H}\right)=\beta_{y0}*y_{j0}$$

μj (Ͳti, γt) = time-averaged mean of yields of farm j using technology Ͳti with climate γt; j = farm index, j= 1, …, J farms in data sample representing the integrated assessment region study area; Ͳti = technology and management used in period t=H or F, adapted to climate i=H or F; γjt = crop yield in year t (kg/ha); βy0 = YH/Y0 = normalization factor used to scale survey data yields to the current period mean; Y0 = mean of observed yields in the survey data for base year t=0; YH = mean of yields averaged over all farms and years in the current period, obtained from secondary data in the survey area.

(3) $$R_{j21}=\rho_{H}*a_{jH}*\mu_{j}\left(T_{HH},\gamma_{H}\right)$$

ajt = total crop area on the farm in period t (ha); Rjqs = time averaged revenue for part q and system s (rupees per farm).

(4) $$C_{j21}=\beta_{c0}*C_{jH}$$

Cjt = production cost for period t (rupees per farm per time); βc0 = CH/C0 = normalization factor used to scale production cost survey data to the current period mean.

(5) $$V_{j21}=R_{j21}-C_{j21}$$

Vjt = Rjt – Cjt = crop net returns for the farm (rupees per time); Vjqs = time-averaged net returns for part q and system s (rupees).

(6) $$\mu_{j}\left(T_{HF},\gamma_{H}\right)=r_{j2}*\mu_{j}\left(T_{HH},\gamma_{H}\right)$$

μj(Ͳti, γt) = time-averaged mean of yields of farm j using technology Ͳti with climate γt; Ͳti = technology and management used in period t=H or F, adapted to climate i=H or F; γjt = crop yield in year t (kg/ha); rj2 = sj (ͲHF, γH)/ sj (ͲHH, γH) = relative yield for analysis of part 2; sj(Ͳti, γt) = simulated crop yield for farm j using technology Ͳti with climate γt.

(7) $$R_{j22}=\rho_{H}*a_{jH}*\mu_{j}\left(T_{HF},\gamma_{H}\right)$$

Rjqs = time averaged revenue for part q and system s (rupees per farm); ajt = total crop area on the farm in period t (ha).

(8) Gj22=Cj22Rj22

Gjt = Cjt/ Rjt = production cost relative to revenue (unit free); Gjqs = Cjqs/ Rjqs = time averaged production cost relative to time averaged revenue for part q and system s; Vjt = Rjt – Cjt = crop net returns for the farm (rupees per time); Vjqs = time averaged net returns for part q and system s (rupees); RHO12= correlation between μj(ͲHH, γF) and μj(ͲHH, γH)

(9) $$C_{j22}=G_{j22}*R_{j22}$$

(10) $$V_{j22}=R_{j22}-C_{j22}$$

**Figure 2:**
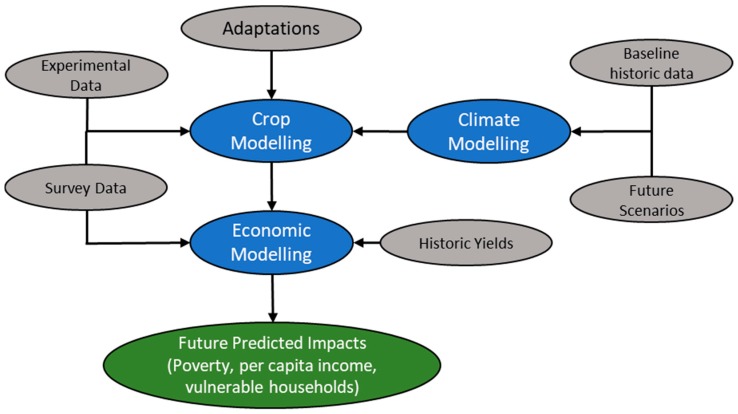
Methodological framework—climate, crop, and economic models’ integration.

The TOA-MD incorporated the statistical correlation between environmental, social, and economic impacts of technology adoption on Net Return (NR), Per Capita Income (PCI), and poverty in simulation. The model simulates the impacts from 0% to 100% of the full range of adoption rates [82]. For a better understanding of the reader, the methodological framework developed by the global AgMIP team followed in this study is presented in Figure 2.

### 2.6. Climate Change Adaptation Package

To overcome the climate impacts, it was observed that expert opinion should be needed for possible climate change adaptation for rice and wheat crops. Adaptation refers to the transition of natural or human systems of response to natural or anticipated climate stimuli or their consequences, moderating harm or exploiting beneficial opportunities [83]. In this study, it was important to understand how crop productivity under a changing climate would be influenced by changes in field management. Therefore, three sessions with experts of agriculture and its allied disciplines and the farmers’ information were recoded, and the probable adaptation packages were modeled with the existing data. The adaptation strategies discussed with stakeholders were focused on increasing levels of application of nitrogen fertilizers, changing plant population, and virtual cultivar. Therefore, this methodology of management was used as the method of adaptation. After careful evaluation and estimation, the adaptation packages for both major crop activities, i.e., rice and wheat in the survey area, were developed. The adaptation package for wheat is given below in Table 1 and for rice in Table 2.

## 3. Results and Discussion

### 3.1. Calibration and Evaluation of DSSAT and APSIM 

CSM-CERES (crop environment resource synthesis) rice and wheat in DSSAT and APSIM-Oryza2000 and wheat were calibrated using field experimental data. The rice experiment was conducted on sowing dates starting from 1 July, 15 July, and 30 July with rice cultivars, i.e., Super Basmati, Basmati-2000, and Basmati-385. The crop models, DSSAT and APSIM, were calibrated starting at 1 July sowing dates and evaluated on 15 July and 30 July for all cultivars. The models showed close agreement between observed and simulated values for rice cultivar Super Basmati (Table 3). Both models showed similar days to anthesis and maturity during the calibration of rice. A good association was recorded between observed and simulated grain yield with an error of 2.9% for DSSAT and 4.9% for APSIM. The crop model evaluation for rice also showed a good result (Table 4). Days to anthesis and maturity were simulated well by DSSAT and APSIM with 1–3 days difference. The crop models simulated the grain yield very well with a root mean square error (RMSE) of 237 kg ha-1 for DSSAT and 202 kg ha-1 for APSIM. The wheat experiment was conducted at various levels of nitrogen (0, 55, 110, and 120 kg ha-1) and wheat cultivars, i.e., Faisalabad-2008, Sehar-2006, and Lasani-2008. The models were calibrated at 110 N kg ha-1 for all cultivars. The calibration results showed a close match between observed and simulated values for all parameters for wheat cultivar Faisalabad-2008 (Table 3).

Crop models simulated days to anthesis and maturity with 1–2 days difference during calibration. The grain yield was simulated well by DSSAT mode with an error of 5.5%, while the higher error of 15% was recorded in APSIM as compared to DSSAT (Table 3). Model evaluation results indicated that both models simulated well the anthesis and the maturity days with errors ranging from 0–0.9% and RMSE of 0.41 to 0.82 days at all nitrogen levels. The grain yield was simulated well by DSSAT at 0 and 55 N kg ha-1 with errors ranging 3–7%, while APSIM showed more errors ranging 13–26%. The APSIM model under-simulated the grain yield at the zero nitrogen level. Overall, crop models calibration and evaluation results for rice-wheat showed a close association and were accepted for further climate change analysis.

### 3.2. Relative Yields of Crops

The TOA-MD model used relative yields estimated as the combination of expected predicted yields and actual farm yields in both CSMs, APSIM and DSSAT, for economic analysis. The relative yields given in Table 5 showed the variation in yield as a result of climate change, either positive or negative. For both major crops in the study area, i.e., rice and wheat, the relative yield showed a declining yield pattern for both crop models, APSIM and DSSAT, considering all five GCMs [60]. The rice crop is more influenced by climatic changes as compared to wheat due to its vulnerability to rising temperature and lack of water supply as per the needed quantity, as the rising temperature and the growing concentrations of carbon provide a buffer for the wheat [47].

### 3.3. Climate Change Impact Assessment

**Figure 3:**
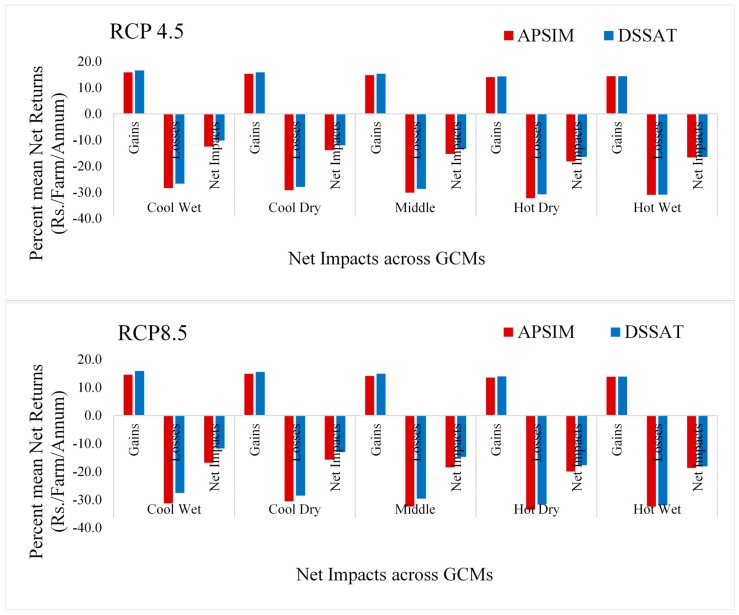
Climate change impacts on the current integrated agricultural production system.

For climate change impact assessment, APSIM and DSSAT crop models were used for five GCMs under RCP 4.5 and 8.5. The results using APSIM showed that gains ranged from 14% to 15.9%, and losses ranged from 28.4% to 32.2% for RCP 4.5. Under RCP 8.5, gains varied from 13.6% to 14.5%, and losses ranged from 30.6% to 33.6%. The same pattern was also observed using DSSAT, where gains in RCP 4.5 ranged from 14.3% to 16.5% and losses from 26.7% to 30.9%. Under RCP 8.5, gains ranged from 13.9% to 15.9%, and losses ranged from 27.6% to 32.0%. Figure 3 showed the gains and the losses as well as the net impact of climate change under both RCPs; crop simulation models were negative, and these results are in line with the previous studies [60,84,85].

The variation in results between DSSAT and APSIM could be due to different assumption parameters. The APSIM model showed more effect of climate change as compared to DSSAT. The model empirically calculates the mean crown temperature to determine the thermal time from daily maximum and minimum temperatures and calculates temperature stress by daily mean temperature. A good model integrates all crop parameters and the effect of stresses on these parameters for final grain yield [86]. Liu et al. [87] also pointed to the need to improve the heat response of APSIM-wheat.

The household vulnerability under RCP 4.5 ranged from 74.2% to 82.4% and 70.2% to 80.3% for APSIM and DSSAT, respectively. Results found household vulnerability was higher with hot dry conditions in GCM and lower with cool wet climate conditions in GCM [8,85]. The reported net returns were RS 644,080 per farm with RS 100,924 per capita income without climate change. However, for crop simulation model APSIM, net returns and per capita income with climate change ranged from RS 489,585 per farm to RS 535,793 per farm and RS 77,378 to RS 84,398, respectively, for RCP 4.5. The net returns for DSSAT ranged from RS 503,041 per farm to RS 555,207 per farm and per capita income from RS 79,289 to RS 87,467 for RCP 4.5. The overall results of the economic impacts of climate change on the household are presented in Table 6 using RCP 4.5.

Table 7 shows the climate change impact on socio-economic factors in aggregate data for RCP 8.5. The susceptibility of RCP 8.5 to climate change for households in cumulative results ranged from 79.0% to 84.4% for APSIM and from 72.9% to 82.4% for DSSAT. The net returns per farm and per capita income with climate change effect ranged from RS 475,905 to RS 509,203 and from RS 75,297 to RS 80,391 for APSIM. Both socioeconomic indicators net returns per farm and per capita income for DSSAT varied between RS 490,489 to RS 542,314 and RS 77,453 to RS 85,435, respectively. 

**Figure 4:**
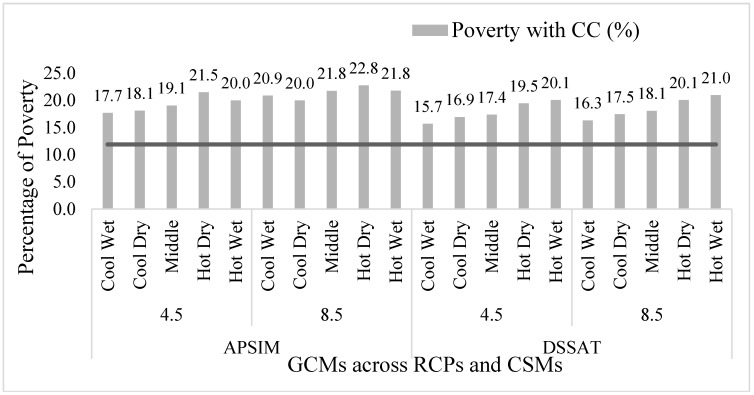
Impact of climate change in the current rice wheat cropping system on poverty.

The poverty reported without climate change was 11.9 %, while climate change poverty in both plant simulation models varied depending on climate conditions. The poverty rate in RCP 8.5 was slightly higher than in RCP 4.5 in all crop modeling simulations [8,60]. Poverty in RCP 4.5 ranged from 17.7% to 21.5% in APSIM and from 15.7% to 20.1% in DSSAT. Poverty in RCP 8.5, however, ranged from 20.0% to 22.8% in APSIM to 16.3% to 21.0% in DSSAT (Figure 4). The highest poverty observed was for GCM hot dry in the crop simulation model APSIM [8].

### 3.4. Impact of Adaptation Package in RWCS

Table 8 shows the adaptation benefits of the current agricultural system using both DSSAT and APSIM crop simulation models. The RWCS adoption rate for crop simulation model DSSAT was about 78%, and for the second crop simulation model (APSIM), it was about 68.34%. The observed net returns, the per capita income, and the poverty for the study area without adaptation were RS 644,080 per farm and RS 100,924, and 11.9%, respectively. 

However, net returns with adaptation were RS 837,267 per farm for DSSAT and RS 775,011 per farm for APSIM. The per capita income of the survey area for DSSAT was RS 129,445 and RS 121,191 for APSIM with adaptation. As far as poverty is concerned, it reduced to 6.31% using DSSAT and 6.61% for APSIM because of the introduced adaptations.

Comparison of results by both crop simulation models (DSSAT and APSIM) showed that, in DSSAT, all indicators were more positive than APSIM’s adoption rate, net returns, per capita income, and poverty [61,88]. This means that DSSAT is more sensitive than APSIM to the adaptation package added. 

**Figure 5:**
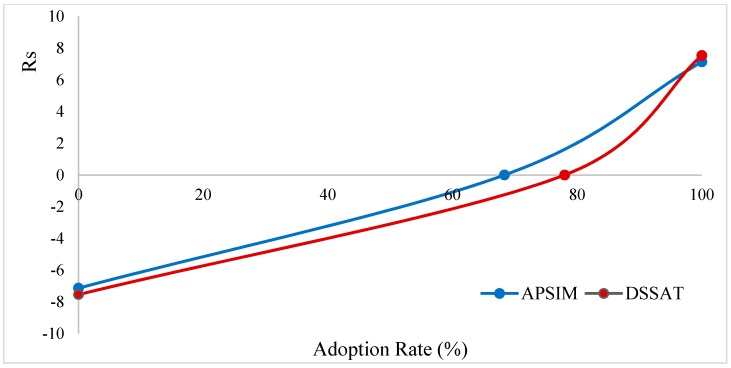
Adoption curve of climate change adaptation using crop models.

Figure 5 shows the adoption curves for the farming system being observed as an aggregate for both DSSAT and APSIM simulation models. APSIM’s adoption rate was around 68%, while DSSAT’s adoption rate was around 78%. 

## 4. Conclusions

In this paper, we focused on the intention of adaptation strategies adopted by the farmers and its impact on household poverty in the integrated rice-wheat agricultural production system of Pakistan. Climate risks such as water stress and increased incidences of pests and diseases are present in the study area. This study mainly focused on the current adaptation strategies that can benefit the farmers by adopting some farm-level management. Therefore, the study was beyond the limits of future adaptation strategies, e.g., insect pest management discussed earlier. These adaptations can be taken as future research areas and the limitation of the present study. 

The comparison of observed climate parameters in the year of study with medium-term patterns, showed that rice and wheat yields were both negatively affected, indicating the risk of production and the limited capacity of farmers to adapt within the season. Climate change is a persistent and pervasive threat to traditional agricultural practices in farming communities. There is no other statement but that Pakistan is one of the countries most affected by climate change. Due to limited financial and farm mechanization, the harmful impacts of climate change are rising. The declining trend in relative yields from crop modeling showed that climate factors such as erratic rainfall and rising temperatures have negative effects on agricultural productivity [8,60,85,88].

The different responses between APSIM and DSSAT provide an insight into how the crop models respond differently to combinations of temperature, precipitation, and interaction with the use of crop management practices used in the study field. DSSAT, for example, shows that the effects of climate change on yields across the various GCMs are relatively small compared to APSIM, which shows large negative impacts. The causes of these differences need to be further studied, but this illustrates the importance of using a multi-model approach to capture the spectrum of uncertainty that comes from the models and the data.

The first part of the study was an impact assessment on climate change that claimed it would adversely affect the area’s agricultural production. The second part stressed the importance of adaptation approaches after evaluating the effect on the study area. The proposed adaptation package, such as an increase in the sowing density, improved method of fertilizer application (fertigation) and improved cultivars for wheat. Similarly, for rice, an increase in the sowing density, the fertigation, and the nitrogen application would lead farmers to cater to the adverse impacts of climate change in the study area. Our findings suggest the importance of these adaptation strategies in Pakistan, and generally in South Asia, aimed at increasing the resistance of cereal farmers.

## Figures and Tables

**Table 1:** Climate change adaptation package used for wheat.

Parameter / Variable	Base Value (S-1)	Units	Crop Model (CM)	CM- ID	Describe Change	Value (S-2)
Improved fertilizer method	Broadcast	-	APSIM and DSSAT	AP002	Applied with irrigation water	-
Sowing density	330	No. per m2	APSIM and DSSAT	Plpop	Increase in plant population by 10%	363
Climate resilient cultivar	-	-	APSIM and DSSAT	-	Genetically modified cultivar	-

Source: author’s finding. APSIM: Agricultural Production Systems Simulator; DSSAT: Decision Support System for Agrotechnology Transfer.

**Table 2:** Climate change adaptation package used for rice.

Parameter / Variable	Base Value (S-1)	Units	Crop Model (CM)	CM- ID	Describe Change	Value (S-2)
Improved fertilizer method	Broadcast	-	APSIM and DSSAT	AP002	Applied with irrigation water	-
Sowing density	25	No. per hill	APSIM and DSSAT	Plpop	10% increase in plant population	28
Increase in nitrogen (recommended concentration)	97	kg/ha	APSIM and DSSAT	156	Recommended nitrogen dose is compulsory	-

Source: author’s finding.

**Table 3:** Calibration of DSSAT and APSIM for rice-wheat at various parameters using field.

Rice	DSSAT			APSIM		
**Parameters**	**Obs.**	**Sim.**	**% Error**	**Obs.**	**Sim.**	**% Error**
Days to anthesis (days)	62	62	0.00	62	62	0.00
Days to maturity (days)	98	98	0.00	98	98	1.02
Grain yield (kg ha^-1^)	4828	4686	2.94	4828	4686	4.99
Biological yield (kg ha^-1^)	11881	11690	1.61	11881	11690	9.74
**Wheat**						
Days to anthesis (days)	110	109	0.91	110	110	0.00
Days to maturity (days)	141	141	0.00	141	145	–2.84
Grain yield (kg ha^-1^)	4136	4366	–5.56	4136	4774	–15.43
Biological yield (kg ha^-1^)	10343	10398	–0.53	10343	11972	–15.75

Obs.: observation; Sim.: simulation.

**Table 4:** Percent error during the evaluation of DSSAT and APSIM at various parameters using field experimental data.

**Rice**								
**Parameters**	**Days to anthesis**		**Days to maturity**		**Grain yield**		**Biological yield**	
Models	DSSAT	APSIM	DSSAT	APSIM	DSSAT	APSIM	DSSAT	APSIM
15 July	–3.22	6.45	–4.04	0	4.45	0.007	1.07	0.62
30 July	1.69	5.08	–0.99	1.98	5.89	7.4	2.8	2.1
RMSE	1.58 day	3.54 day	2.91 day	1.41 day	237 kg ha^-1^	202 kg ha^-1^	247 kg ha^-1^	230 kg ha^-1^
**Wheat**								
0 N kg ha^-1^	0	0.94	0.71	1.42	3	–26.4	2.3	29.91
55 N kg ha^-1^	0	0.94	0	0.7	6.9	–13.2	–1.7	18.25
120 N kg ha^-1^	–0.93	0	–0.7	0	–0.9	–7.2	–1.4	3.71
RMSE	0.58 day	0.41 day	0.82 day	0.61 day	176 kg ha^-1^	293 kg ha^-1^	278 kg ha^-1^	868 kg ha^-1^

RMSE: root mean square error.

**Table 5:** Relative yields distribution of modeled crops for current climatic conditions.

Crops	RCPs	Global Circulation Models (GCMs)				
		Cool Wet	Cool Dry	Middle	Hot Dry	Hot Wet
Rice	APSIM_4.5	0.75	0.74	0.71	0.67	0.68
	APSIM_8.5	0.70	0.71	0.66	0.64	0.65
	DSSAT_4.5	0.83	0.79	0.76	0.72	0.71
	DSSAT_8.5	0.81	0.77	0.74	0.70	0.69
Wheat	APSIM_4.5	0.97	0.98	0.97	0.96	0.96
	APSIM_8.5	0.96	0.94	0.94	0.92	0.93
	DSSAT_4.5	0.98	0.99	0.97	0.98	0.97
	DSSAT_8.5	0.97	0.95	0.96	0.94	0.94

RCPs: representative concentration pathways.

**Table 6:** Climate change impact on socio-economic factors in aggregate data for RCP 4.5.

CM	GCM	Vulnerable Farm Household (%)	NR with CC	PCI with CC
APSIM	Cool Wet	74.2	535,793	84,398
	Cool Dry	76.4	524,683	82,759
	Middle	78.5	513,107	80,933
	Hot Dry	82.4	489,585	77,378
	Hot Wet	80.5	502,071	79,132
DSSAT	Cool Wet	70.2	555,207	87,467
	Cool Dry	73.4	539,799	84,967
	Middle	75.8	528,406	83,249
	Hot Dry	80.3	503,435	79,358
	Hot Wet	80.3	503,041	79,289

CM: circulation model; CC: climate change; NR: net return; PCI: per capita income.

**Table 7:** Climate change impact on socio-economic factors in aggregate data for RCP 8.5.

CM	GCM	Vulnerable Farm Household (%)	NR with CC	PCI with CC
APSIM	Cool Wet	80.3	500,871	79,027
	Cool Dry	79.0	509,203	80,391
	Middle	82.4	488,209	77,193
	Hot Dry	84.4	475,905	75,297
	Hot Wet	83.1	486,057	76,672
DSSAT	Cool Wet	72.9	542,314	85,435
	Cool Dry	74.9	532,241	83,771
	Middle	77.8	517,433	81,584
	Hot Dry	81.9	493,642	78,038
	Hot Wet	82.4	490,489	77,453

**Table 8:** Impact of climate change adaptations on the current rice-wheat agricultural production system using DSSAT and APSIM.

Crop Models	Adoption Rate (%)	Net Returns (RS/Farm/Annum)	Per Capita Income (RS)	Poverty (%)
DSSAT	77.99	837,267	129,445	6.31
APSIM	68.34	775,011	121,191	6.61

RS: Pakistani rupee.
